# A Novel Automatic Quantification Protocol for Biomarkers of Tauopathies in the Hippocampus and Entorhinal Cortex of Post-Mortem Samples Using an Extended Semi-Siamese U-Net

**DOI:** 10.3390/biology11081131

**Published:** 2022-07-28

**Authors:** Luis A. Campero-Garcia, Jose A. Cantoral-Ceballos, Alejandra Martinez-Maldonado, Jose Luna-Muñoz, Miguel A. Ontiveros-Torres, Andres E. Gutierrez-Rodriguez

**Affiliations:** 1 School of Engineering and Sciences, Tecnologico de Monterrey, Monterrey 64849, Mexico; luis.acg@outlook.de (L.A.C.-G.); joseantonio.cantoral@tec.mx (J.A.C.-C.); 2 Health Sciences Faculty, Universidad Anahuac Mexico Norte, Mexico City 52786, Mexico; alejandra.martinezma@anahuac.mx; 3 Faculty of Higher Studies Cuautitlan, Biological Sciences, National Dementia BioBank, UNAM, Mexico City 04510, Mexico; jluna_tau67@comunidad.unam.mx; 4 National Brain Bank-UNPHU, Universidad Nacional Pedro Henríquez Ureña, Santo Domingo 1423, Dominican Republic; 5 Institute for the Future of Education, Tecnologico de Monterrey, Monterrey 64849, Mexico

**Keywords:** biomarkers, convolutional neural networks, deep learning, immunofluorescence quantification, dementias, tau protein, U-Net

## Abstract

**Simple Summary:**

Tauopathies is a term coined to describe an umbrella of disorders characterized by abnormal Tau polypeptide deposits in neurons, glial cells, and extracellular space. In this work, we propose a novel quantification protocol for the study of tauopathies based on the U-Net neural network architecture. We also compare the proposed method against other state of the art variations of the U-Net to test its efficacy.

**Abstract:**

Efforts have been made to diagnose and predict the course of different neurodegenerative diseases through various imaging techniques. Particularly tauopathies, where the tau polypeptide is a key participant in molecular pathogenesis, have significantly increased their morbidity and mortality in the human population over the years. However, the standard approach to exploring the phenomenon of neurodegeneration in tauopathies has not been directed at understanding the molecular mechanism that causes the aberrant polymeric and fibrillar behavior of the tau protein, which forms neurofibrillary tangles that replace neuronal populations in the hippocampal and cortical regions. The main objective of this work is to implement a novel quantification protocol for different biomarkers based on pathological post-translational modifications undergone by tau in the brains of patients with tauopathies. The quantification protocol consists of an adaptation of the U-Net neural network architecture. We used the resulting segmentation masks for the quantification of combined fluorescent signals of the different molecular changes tau underwent in neurofibrillary tangles. The quantification considers the neurofibrillary tangles as an individual study structure separated from the rest of the quadrant present in the images. This allows us to detect unconventional interaction signals between the different biomarkers. Our algorithm provides information that will be fundamental to understanding the pathogenesis of dementias with another computational analysis approach in subsequent studies.

## 1. Introduction

Neurodegenerative diseases, known as tauopathies, are proteinopathies whose main characteristic is the formation of insoluble fibrillar protein structures called neurofibrillary tangles (NFTs) that are formed mainly by abnormal chemical species of the tau protein in various regions of the human brain.

This group of diseases includes Alzheimer’s disease (AD), frontotemporal dementia (FTD), progressive supranuclear palsy (PSP), and tangle only (TO) [1,2,3]. In these pathologies, the tau polypeptide undergoes several abnormal post-translational modifications (PTMs) such as hyperphosphorylation, proteolysis, and conformational changes, among others. These chemical and physical changes lead the protein to dissociate from the microtubules to polymerize forming insoluble aggregates called paired helical filaments (PHFs), which will assemble the NFTs [4].

Studies using antibodies directed against the different PTMs of the tau protein have begun to use histological and triple-label immunofluorescence in the differential diagnosis of tauopathies [5]. However, the PTMs against which these antibodies are directed have only been studied qualitatively at a molecular level in the brain, where the disease occurs. Post-mortem brain tissues from patients with tauopathies, in addition to confirming the diagnosis, can support the study of the mechanisms that trigger the aberrant behavior of the tau polypeptide and culminate in the death of the neuronal population [6].

The fluorescent signals of the immunostaining against these molecular events are valuable for quantifying the phenomena of neurodegeneration in this group of diseases. Therefore, our study focuses on designing a new protocol for obtaining quantitative information on the pathogenesis of tauopathies through image segmentation using deep learning (DL). This quantification is performed based on tau polypeptide biomarkers detected in NFTs by three-signal immunofluorescence techniques.

Common unsupervised methods for segmentation of biomedical images include K-means clustering and thresholding [7,8]. However, they might fail in complex scenarios like the segmentation of brain tumors, where thresholding failed to provide consistent outputs, whilst DL approaches have achieved better results [9]. K-means also suffers from a lack of consistent outcomes and might need human validation due to its unsupervised nature [8]. DL models, particularly convolutional neural networks (CNNs), have proven to be effective tools for the segmentation of a wide range of biomedical images [10,11,12,13]. The U-Net architecture [14] has been widely used used in these cases. This is because an important challenge in these applications is the ability to work with small datasets and with a limited amount of annotated samples, since generating additional samples is expensive and requires domain expertise [15,16,17], and the U-Net was designed with that in mind. Many variants have been proposed to fine-tune it for different applications [18]. Recent applications of the U-Net include the semi-Siamese U-Net [13], which was proposed to separate lung and heart bioimpedance images through two parallel decoders, and the RCU-Net [12], which was used to segment breast tumors in ultrasound images with the use of residual and dense blocks. The semi-Siamese U-Net outperformed the traditional U-Net by 2.19% in the DICE coefficient, while the RCU-Net outperformed it by 2.01%, each one in their respective tasks.

The RandomSURF algorithm is within the protocols that have been used for the quantification of amyloid beta (β-Amyloid) peptide plaques, which is another major protein in the pathogenesis of AD [19]. The quantification was performed through the segmentation of a fluorescent signal in mouse brains expressing the mutated genes of familial AD. After segmentation, quantification is obtained by the number of segmented pixels. Although ImageSURF delivered good results, the images used in the study were limited to a single β-Amyloid fluorescence signal (excluding the NFTs generated by the tau polypeptide) with low resolution, thus some structures are not available for segmentation and subsequent fluorescence quantification.

In addition, the artificial concentration of β-Amyloid in mouse brains may not correlate at all to what occurs in the late stages of AD in human brains. Indeed, it has been shown that one of the factors limiting our understanding of human neurological diseases lies in the inherent limits of animal models [20]. Furthermore, a great amount of research on the quantification of β-Amyloid and tau polypeptides is mainly aimed at non-invasive clinical diagnosis of neurodegeneration [21,22,23].

For the development and validation of the fluorescent signal quantification protocol, we used a set of four types of tauopathy images to build a training dataset of adequate size: AD, PSP, TO, and FTD. In all four disease types, the images were obtained by three-signal immunofluorescence assays. Although the biological interpretation of fluorescence in each disease varies, our approach allows us to consider them based on the use of biomarkers against abnormal tau polypeptide PTMs in all cases.

As part of our study, we propose a novel model based on the semi-Siamese U-Net [13]. We compare its performance to a four-output modified version of the U-Net [14] and three other state-of-the-art neural networks: the RCU-Net [12] including their proposed dense block, the RCU-Net without the dense block (Res U-Net), and the Inception U-Net [18].

The proposed method outperformed the other methods in our benchmark in the four metrics used to assess performance, achieving an intersection over union score of 82.68%, a DICE coefficient of 90.64%, a false positive rate of 4.37%, and a true positive rate of 86.9%.

The main contributions of this paper are, on one side, the design and validation of a quantification protocol based on DL fluorescent image segmentation for tau polypeptide biomarkers obtained from brain tissue of patients with different tauopathies. On the other side, this work expands the state-of-the-art by proposing a novel U-Net-based neural network model.

The remainder of this paper is organized as follows. We begin by providing details on how the data were obtained, and also by describing our segmentation protocol, its components, and the experiments that were carried out to test the different models in Section 2. Section 3 presents the results of our experiments. Finally, the implications of the results are discussed in Section 4, whilst conclusions and final remarks are provided in Section 5.

## 2. Materials and Methods

### 2.1. Data Acquisition

**Figure 1:**
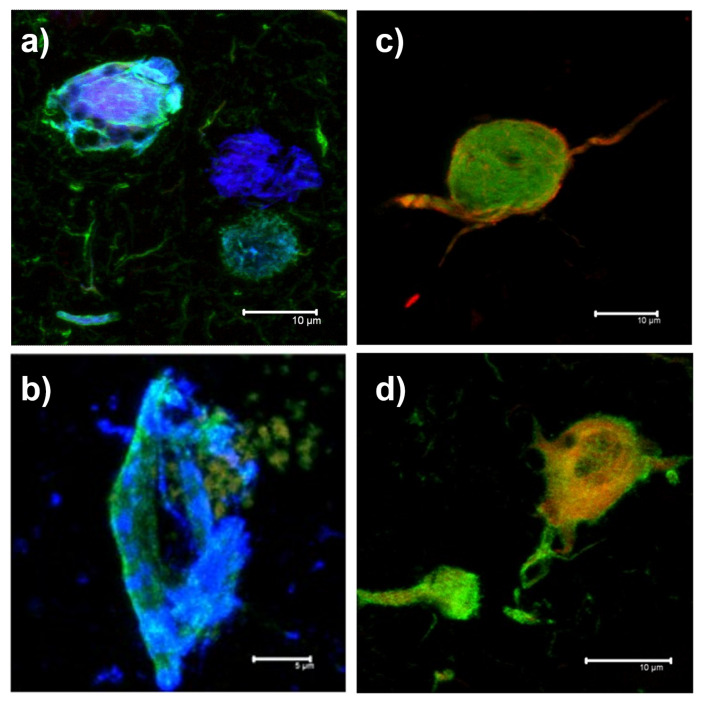
**Representative examples of the images obtained by Immunofluorescence for each of the proteinopathies-tauopathies in the hippocampus of the clinical cases included in this study.** High magnifications (100×) are observed for neurofibrillary tangles for each disease group. (**a**) Alzheimer’s disease; (**b**) progressive supranuclear palsy; (**c**) taEngle only; (**d**) frontotemporal dementia.

The image database for this research was obtained from the brains of patients with different tauopathies and was facilitated by a collaborative project between the National Dementia Biobank and the School of Engineering and Sciences of Tecnologico de Monterrey. These immunofluorescence images correspond to NFTs, visualized using secondary antibodies coupled to fluorochromes and primary antibodies against epitopes corresponding to different pathological PTMs undergone by the tau polypeptide in four types of proteinopathy-tauopathies: AD, PSP, TO, and FTD (Figure 1). The images, obtained at 100× magnification, were processed with three fluorescent signals, each signal corresponding to a different antibody or molecule with specific binding to polymeric insoluble fibrillar forms such as thiazine red (TR), used to specifically stain MNFs.

**Figure 2:**
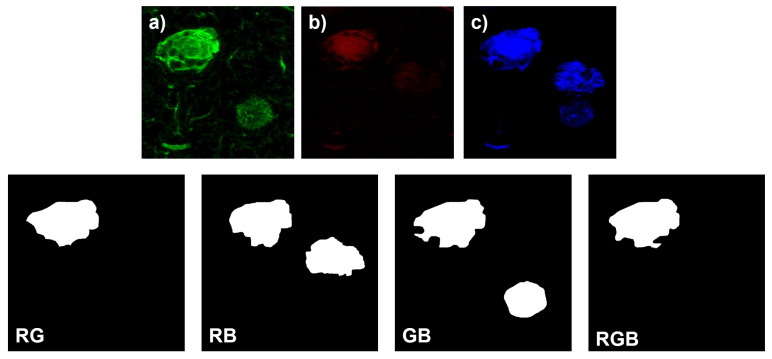
**Representative images of an immunofluorescence assay with double immunostaining and thiazine red dye staining of the hippocampus of an AD patient and examples of assignment of the corresponding training labels.** (**a**) Immunoreactivity of AT8 antibody (green channel) detecting pathological phosphorylations at amino acids serine 202 and threonine 205 of the tau protein is observed. (**b**) Positive staining against fibrillar forms of tau protein is observed in the red channel. (**c**) The 396 antibody (blue channel) detects pathological phosphorylation at serine 396 of the tau protein. The images at the bottom are the training labels. The labels show the specific channel combination that they are targeting. R: red; G: green; B: blue.

Several combination protocols were used for immunostaining of the different PTMs in the brain regions, so the biological and molecular interpretation of the results must take into account the combination method used as the immunostaining mechanism, as well as the specific tauopathy in each case. Figure 2 shows an example of the combination of individual channels.

For each channel, the samples were immunostained with the following antibodies, and the epitopes against the pathological PTMs in the tau polypeptide are shown:

- **Red Channel:** Thiazine red and the antibodies TG3 (regional conformational change with phosphorylation at amino acid threonine 231), pT231 (phosphorylation at threonine 231), Alz50 (structural conformational change), pS396 (phosphorylation at serine 396), and AT100 (regional conformational change and phosphorylation at serine 202, threonine 205, threonine 212, and serine 214).
- **Green channel:** AT8 (phosphorylation at serine 202, threonine 205 and serine 208), CP13 (phosphorylation at serine 202), 499 (amino terminal end), Tau-7 (carboxyl terminal end), TauC3 (proteolysis at aspartic 421 carboxyl terminal end) antibodies, PHF1 (phosphorylation at serines 396 and 404), AD2 (phosphorylation at amino acids serine 396 and serine 404), and 423 (proteolysis at glutamic 391 of the carboxyl-terminal end).
- **Blue Channel:** Antibodies pS396, S-199 (phosphorylation at amino acid serine 199), pT231, and Alz50.

After immunostaining, images were captured using the confocal microscopy technique (SP8 Leica). The physical size of the captured samples ranges from 26.97 μm to 134.85 μm on each side, with a digital resolution of 512 × 512 pixels in all cases. The final dataset contains a total of 97 images where 19 correspond to AD, 51 to PSP, 10 to TO, and 17 to FTD. The obtention of images of this nature is difficult, as they are taken from the brains of deceased dementia patients. Therefore, the ability of the U-Net to learn from relatively few examples is vital.

### 2.2. Manual Segmentation of Biomarker Signals

In order to obtain the ground truth, the training images were manually segmented using the online application Labelbox (https://labelbox.com/, accessed on 28 May 2022). The results were supervised and validated by three experts in neurophysiology and molecular pathogenesis of neurodegenerative diseases with experience in obtaining and analyzing immunofluorescent images. In addition, two computational engineering researchers with expertise in neural networks with biomedical images further validated the annotations.

For each of the available images, four labels were generated to consider all color channel combinations: red and green (RG); red and blue (RB); green and blue (GB); red, green, and blue (RGB). For the region to be included in the segmentation, it must be part of the central growth nucleus of the NFT corresponding to the fluorescent signal and it must show significant intensity in all respective channels. For the final segmentation to be accepted, the experts must agree. If they did not agree, a final segmentation was obtained by mutual consultation. The manual measurements were stored as binary segmentation masks in JPEG format. To access the information for each label, the binary segmentation masks were converted into NumPy arrays and then processed using the Python programming language.

### 2.3. Network Architecture

The proposed architecture is based on the U-Net, which has become prominent in the field of medical image segmentation for its capacity to obtain good results with few labeled images [14]. The original U-Net consists of an encoder path (left-side) and a decoder path (right-side), with skip connections between them to transfer the corresponding feature map at each level of the network from the encoder to the decoder. The characteristics of the U-Net apply to our study, where the aim is to segregate the different affected regions.

**Figure 3:**
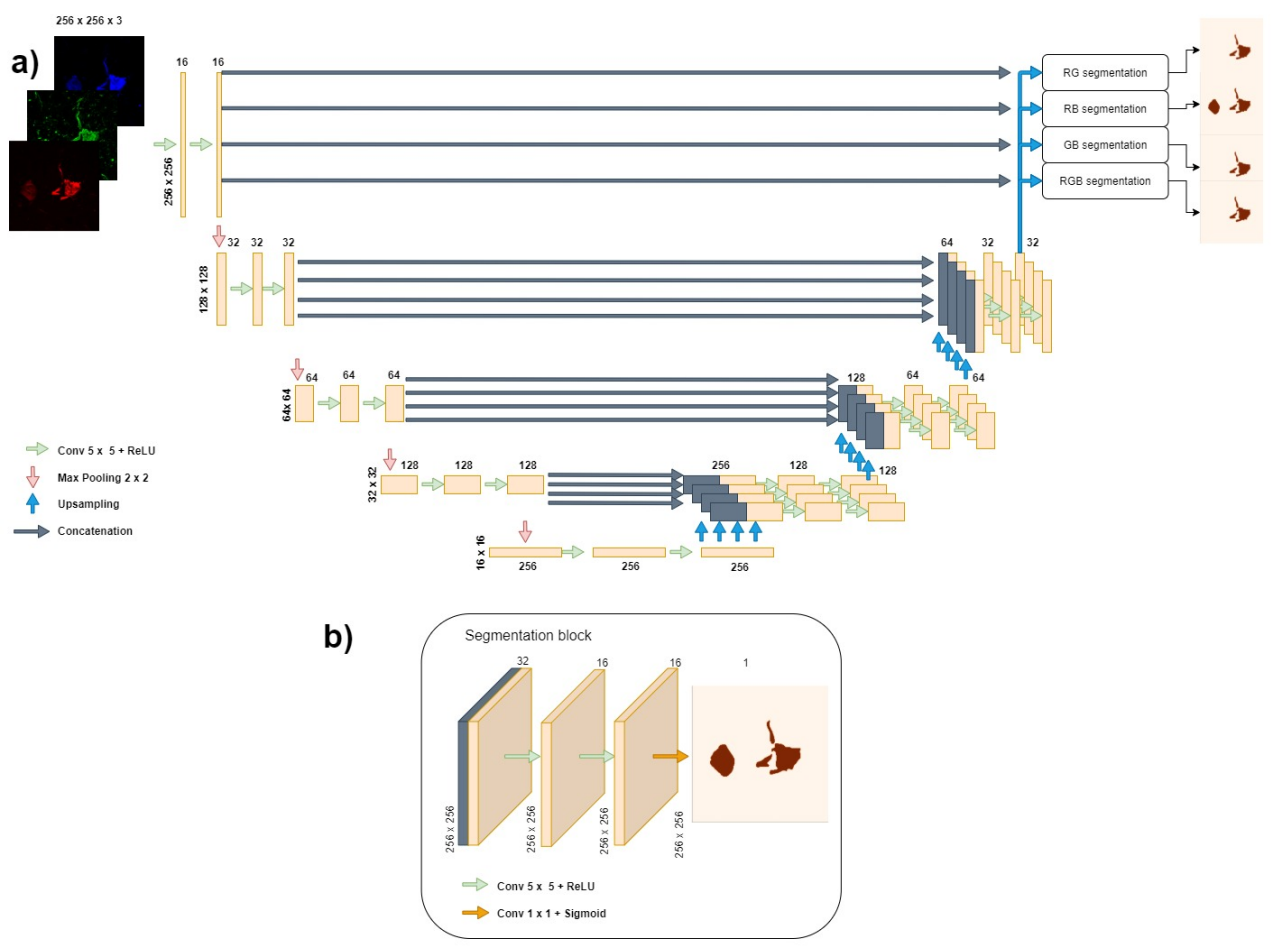
**Proposed model of the extended semi-Siamese U-Net neural network.** (**a**) The proposed model. Each box corresponds to a multi-channel feature map. The number of channels is denoted at the top of each box; the height and width of each feature map are provided at the edge of each box. The encoder path on the left consists of five levels (including the bottleneck at the bottom), each one consisting of two convolutional layers. The outputs of the encoder are concatenated with the upsampled feature maps in the decoder (gray arrows) before being passed through two additional convolutional layers. Each of the four decoding paths gets its own copy of the encoder feature map corresponding to its level. At the end of the network, there are four segmentation blocks. (**b**) The final segmentation block. At the end of the network, a 1×1 convolution is applied to obtain the final segmentation mask.

In this study, we extend the semi-Siamese U-Net [13] to perform the simultaneous segmentation of four different combinations of immunofluorescence channels in dementia images. This novel architecture, shown in Figure 3, consists of one contracting path and four parallel expanding paths to perform the multi-task image segmentation. Each segmentation task shares the parameters of the contracting path. The mapping from image to segmentation masks takes a three-channel 256 × 256 pixel image as the input and then outputs four one-channel 256 × 256 pixel masks. Each level in the encoder consists of two convolutional layers: the first level contains 16 filters at each layer, the second level 32, the third level 64, the fourth level 128, and the bottleneck level 256. Each filter has a size of 5 × 5 and they are applied with "same" padding with a stride of 1. Additionally, a dropout layer with a rate of 0.2 was added after each max-pooling to improve the generalization of the model. In the decoder, upsampling is carried out with the nearest type interpolation to double the size of the encoded feature maps at each level. Then, two convolutional layers, with the same characteristics as in the encoder, are applied, followed by a dropout layer, again using a dropout rate of 0.2. All the convolutional layers use the rectified linear unit (ReLU) activation function, except for the final layer in each output, which uses the sigmoid activation to perform the segmentation with a 1 × 1 convolution.

### 2.4. Evaluation Criteria

To provide quantitative performance comparisons across the five tested models, the experimental results were evaluated using four metrics: true positive (TP), false positive (FP), the Dice coefficient (DC) [24], and the intersection over union (IOU, also called Jaccard index) [25]. These measures are defined in terms of the ground truth (GT) and the segmentation results (SR) obtained from the forward pass of the method to be evaluated.

The TP (Equation (1)) is the proportion of pixels correctly assigned as part of the segmentation to the GT. The higher the TP value, the greater the coverage of the target region.

(1) $$TP=\frac{GT\cap SR}{GT}$$

The FP (Equation (2)) is the proportion of pixels wrongly predicted as part of the segmentation to the GT. The lower the FP, the fewer background pixels that are classified as part of the affected region.

(2) $$FP=\frac{GT\cup SR-GT}{GT}$$

The DC (Equation (3)) is used in segmentation tasks to give a measure of how similar the segmented regions are to the GT. The closer the DC to 1, the more accurate the segmentation result.

(3) $$DC=2\frac{GT\cap SR}{GT+SR}$$

The IOU (Equation (4)) is similar to the DC, but it measures the total overlap of the SR and the GT. The closer the IOU is to 1, the more the overlap of both regions approaches an exact match.

(4) $$IOU=\frac{\left|GT\cap SR\right|}{\left|GT\cup SR\right|}$$

### 2.5. Training Methods and Experimental Design

To get a reliable final model, 5-fold cross-validation was applied in the experiment. The total of 97 images were randomly divided into 5 cases. For each case, 78 images were used for training (80%), and the remaining 19 images were used for validation (20%). The training set in each fold was subjected to the same transformations to augment the available data further. First, a random rotation of 90, 180, or 270 degrees was applied to each image. Then, random elastic deformations [26] were applied to both the original and the rotated image, resulting in 312 images for training after the augmentation. In order to help improve training and inference times, the images and masks were resized to 256×256 pixels using the OpenCV Python library.

We used the Tensorflow v2.6.0 Python API to implement and train all models on a machine with a Ryzen 73,800× processor, 32 GB of RAM, and an NVIDIA GeForce RTX 3070 GPU. On the first fold for each model, we initialized the weights with TensorFlow’s Glorot Uniform initializer with limit values −1 to 1, and stored the values on separate files, then we ran the following folds with the same initial weights.

The experiments were performed using the Adam optimization with learning rates of 0.1, 0.01, 0.001, 0.0005, and 0.0001, obtaining the best performance with a learning rate α=0.0005, using a random test set. Thus, all further models were trained with the Adam optimizer for 50 epochs with a mini-batch size of 4.

Since the training labels consist of binary masks, we used binary cross-entropy to compute our model’s cost function. Equation (5) shows the cross-entropy function across a batch of *n* samples, where *X* is the original image, *Y* is the ground truth label, $\hat{Y}$ is the predicted mask by a trained model, $y_{px}$ is a pixel in the ground truth label, and ${\hat{y}}_{px}$ is a pixel in the predicted segmentation.

(5) $$L\left(X,Y,\hat{Y}\right)=\frac{1}{n}\sum_{px\in X}-\left(y_{px}\log\left({\hat{y}}_{px}\right)+\left(1-y_{px}\right)\log\left(1-{\hat{y}}_{px}\right)\right)$$

Then, the five models were trained using 5-fold cross-validation. Each model used the same training and test samples for each fold. We used the sigmoid activation function in the last segmentation layer to obtain a probability map, applying a threshold of 0.5 to each pixel (i.e., if the pixel’s value was greater than 0.5, then it was included in the final segmentation and used to evaluate performance).

## 3. Results

For each model, five sets of numerical weights were generated. The testing images were input into each model and, for each one, four segmentation masks were obtained. The output images, along with the training labels, were used to calculate the metrics along the four outputs, and then these measurements were averaged to obtain the final results per model using each set of weights. Table 1 summarizes the mean and standard deviation of the four metrics, along with the four outputs of the models.

**Figure 4:**
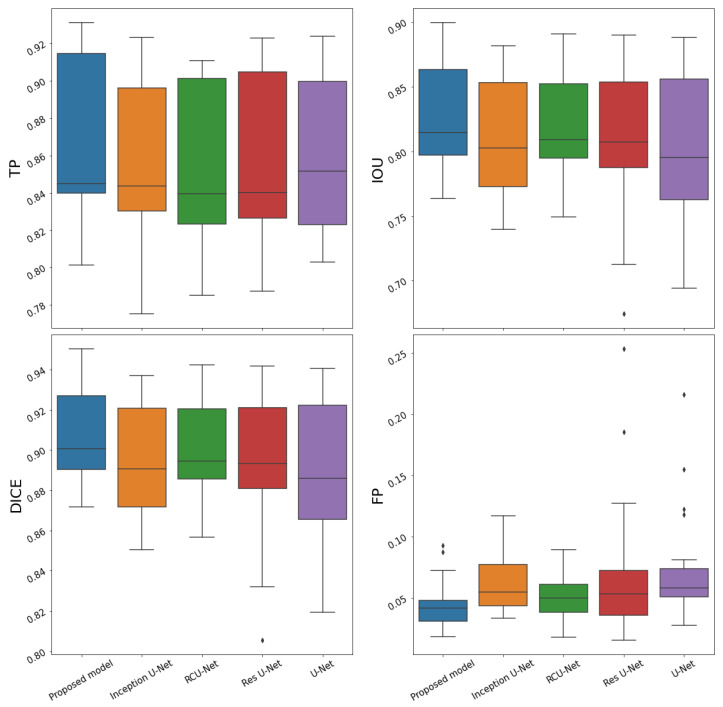
**Performance of the five models in the four metrics.** TP, IOU, DC, and FP averaged across the four outputs using 5-fold cross-validation. The *x*-axis represents the model name and the *y*-axis the values of the metric. It can be seen that the proposed model has a better average performance in all metrics except the TP.

Figure 4 shows the boxplots corresponding to the performance of each model across the four outputs. It can be seen that the proposed model had a better average performance in all the metrics. In comparison to the traditional U-Net, the DC, FP, IOU, and TP of the proposed model improved by 1.7%, 3.12%, 2.43%, and 0.72%, respectively. Additionally, it can be seen that the proposed model is slightly more stable (i.e., has a lower standard deviation) than the models that include a residual path, which is often used to improve stability [18].

**Figure 5:**
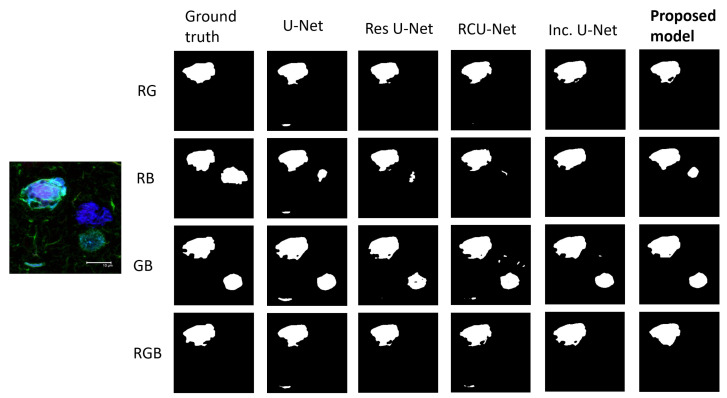
**Experimental segmentation results obtained with the different neural network models.** Each column represents the outputs of the corresponding model and each row one combination of channels. R: red; G: green; B: blue.

In Figure 5, we randomly selected a representative AD image from the test set with different regions highlighted across each color channel combination to show the segmentation capabilities of each model on the desired regions. While the performance metrics across models were close, it can be seen in Figure 5 that the proposed model does a better job including all the desired areas through all four outputs in the segmentation while avoiding the undesired ones, as specified by the ground truth labels.

**Figure 6:**
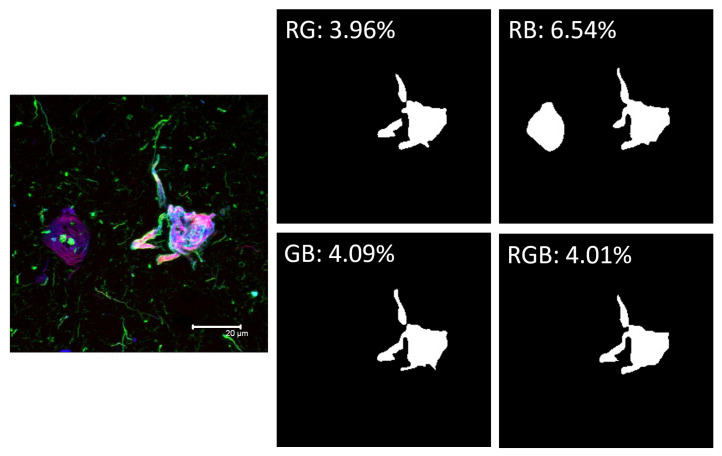
**Quantification of the fluorescent signal using the proposed model.** The quantification consists of counting the number of pixels in the segmented regions and dividing that number by 65,536, the total pixel count in a 256 × 256 pixel image. R: red; G: green; B: blue.

An example of fluorescence quantification can be seen in Figure 6. For each color channel combination, the proposed model generates a binary segmentation mask. Afterward, the total number of white pixels is counted and divided by 65,536, the total number of pixels in the 256 × 256 pixel images, thus obtaining the percentages shown.

## 4. Discussion

In this study, we developed a new protocol for the automatic biomarker quantification of pathological tau polypeptide PTMs and validated our design against four different proteinopathy groups using a semi-Siamese U-Net extension.

The proposed method can localize different types of PTMs occurring in the NFT body and discriminate them from the other pathological events in the quadrant with a DC of 0.9064. Previously, there was no automatic method to obtain quantitative information on fluorescence images that focuses on the particular biological event and biomarker based on the unique structure of the fibrillar lesion. It is important to note that manual segmentation of the four signals in a three-channel image in an experiment requires a time of 15 to 30 min, depending on the complexity of the biological disease events captured in the image. Our design takes only a few seconds to perform the segmentation on a standard CPU.

The segmentation obtained using our DL model, in addition to being faster and simpler, is more reliable, since it is less prone to variability due to the existence of differences in the criteria of human raters, subjectivity that may exist based on these differences, or the quality of the image itself. The proposed method can achieve better performance because each upsampling path can focus on a single task, instead of sharing weights all the way up during the upsampling with the other tasks, like the U-Net, the Inception U-Net, Res U-Net, and the RCU-Net do.

An example of the quantification can be observed in Figure 6. The result offers a higher association of RB channels (thiazine red and pS396). This implies that phosphorylation at serine 396 matures into the polymeric fibrillar form with a value of 6.54%, for being a late event more advanced in the formation of a fibrillar filament than the biomarker for dual phosphorylation at serine 202 and threonine 305 (AT8 antibody), which is a relatively earlier event, with a significantly lower value of 3.96%. These results are congruent with experimental studies that show that in advanced polymeric stages the tau polypeptide is highly phosphorylated at serine 396 [3,27]. This observation highlights the importance of the quantification protocol for understanding the maturational stages of neurofibrillary tau polypeptide changes in different tauopathies and in different brain regions. The quantification would be made based on the unique structure of fibrillar lesions on patients, also including the analysis of other biomarkers controlled by fluorescence.

This work has focused on the development and validation of a computational tool that will be fundamental in our next studies aimed at understanding the pathogenesis and molecular mechanisms of neurodegenerative diseases that trigger chemical changes in the tau protein. This will be carried out in a quantitative and differential way between different channels and their combinations in triple immunostaining experiments. Furthermore, we can use it to quantify and analyze other pathological post-translational events that occur in other important biomarkers, such as β-Amyloid peptide in AD, α-synuclein in Parkinson’s disease, or any event based on immunolabeling or fluorescence.

Finally, we believe that in order to reach an effective early diagnosis for neurodegenerative diseases, it is important to understand the molecular mechanism that leads tau to acquire aberrant and polymeric behavior, which directly impacts the process of neuronal death. Knowing the specific molecular processing will guide us to find biomarkers that can be monitored in less invasive tissues such as cerebrospinal fluid or plasma. For example, the detection of tau phosphorylated in plasma has already been reported [28,29]. Therefore, the use of computational technology such as convolutional neural networks to study this type of post-mortem imaging is very relevant and can provide significant value in the search for specific biomarkers for each group of neurodegenerative diseases, with the aim of implementing an early diagnosis in patients.

## 5. Conclusions

We present a new protocol for the automatic quantification of pathological tau polypeptide PTMs in the hippocampus and validate it in patients affected by different tauopathies. Our design is based on a novel neural network architecture, which was compared with five state-of-the-art U-Net variants, delivering an improvement in performance. The improvement in performance is considered to come from the fact that the network has four decoding paths, and each path can specialize in its segmentation task. This can be seen in our model’s ability to ignore regions that should not be considered at all in the final segmentation. Obtaining quantitative information from post-mortem brain tissue will be fundamental for the study of the pathogenesis of different neurodegenerative diseases explored by immunolabeling and fluorescence techniques, so this quantification protocol will be essential in our future studies with protein biomarkers.

## Figures and Tables

**Table 1:** The mean and the standard deviation of the four metrics across the 4 outputs and the 5-fold cross-validation. The numbers after the ± represent the standard deviation.

Model	DC	FP	IOU	TP
**Proposed model**	**0.9064** ± **0.0223**	**0.0437** ± **0.0201**	**0.8268** ± **0.0402**	**0.8690** ± **0.0424**
Inception U-Net	0.8938 ± 0.028	0.0638 ± 0.0252	0.8092 ± 0.0459	0.8593 ± 0.0408
RCU-Net	0.8984 ± 0.0233	0.0519 ± 0.0202	0.8164 ± 0.0385	0.8536 ± 0.0424
Res U-Net	0.893 ± 0.0335	0.0699 ± 0.0561	0.8083 ± 0.0535	0.8589 ± 0.0439
U-Net	0.8894 ± 0.0333	0.0749 ± 0.0443	0.8025 ± 0.0539	0.8618 ± 0.0396

## Data Availability

The data that support the findings of this study are available from the National Dementia Biobank but restrictions apply to the availability of these data, which were used under license for the current the study, and so are not publicly available. Data are however available from the authors upon reasonable request and with permission of the National Dementia Biobank.

## Abbreviations

The following abbreviations are used in this manuscript:

NFTs	Neurofibrillary Tangles
AD	Alzheimer’s Disease
FTD	Frontotemporal Dementia
PSP	Progressive Supranuclear Palsy
TO	Tangle Only
PTMs	Post-Translational Modifications
PHFs	Paired Helical Filaments
DL	Deep Learning
CNN	Convolutional Neural Networks
TP	True Positive
FP	False Positive
IOU	Intersection Over Union
